# Immunothrombotic Cell–Cell Communication Networks in Coronary Atherosclerosis: Critical Insights from Single-Cell and Spatial Systems Biology

**DOI:** 10.3390/ijms27135900

**Published:** 2026-06-30

**Authors:** Beata Krasińska, Antoni Staniewski, Oliwia Kalus, Joanna Maćkowiak, Zofia Szymańska, Zofia Gramala, Katarzyna Zalewska, Michał Karpiński, Paulina Mertowska, Łucja Rolek, Kinga Koziarska, Krzysztof J. Filipiak, Mansur Rahnama, Mariusz Kowalewski, Calogera Pisano, Giuseppe Maria Raffa, Zbigniew Krasiński, Piotr Suwalski, Vincenzo Nuzzi, Ewelina Grywalska, Tomasz Urbanowicz

**Affiliations:** 1 Department of Hypertensiology, Angiology, and Internal Medicine, Poznan University of Medical Sciences, 61-848 Poznan, Poland; 2 Student Research Group of Cardiology, Poznan University of Medical Sciences, 61-701 Poznan, Poland; 3 Department of Experimental Immunology, Faculty of Medical Sciences, Medical University of Lublin, 20-093 Lublin, Poland; 4 Student Research Group of Experimental Immunology, Medical University of Lublin, 20-093 Lublin, Poland; 5 Central University Hospital, Poznan University of Medical Sciences, 60-352 Poznan, Poland; 6 The Centre of Postgraduate Medical Education, 01-813 Warsaw, Poland; 7 Department of Dental Surgery, Medical University of Lublin, 20-093 Lublin, Poland; 8 Department of Cardiac Surgery, Center of Postgraduate Medical Education, Central Clinical Hospital of the Ministry of Interior, 02-507 Warszawa, Poland; 9 Department of Research, IRCCS ISMETT (Mediterranean Institute for Transplantation and Advanced Specialized Therapies), 90127 Palermo, Italy; 10 Department of Precision Medicine in Medical Surgical and Critical Area (Me.Pre.C.C.), University of Palermo, 90134 Palermo, Italy; 11 Department of Vascular, Endovascular Surgery, Angiology and Phlebology, Poznan University of Medical Science, 61-848 Poznan, Poland; 12 Department of Clinical Cardiology and Heart Failure, IRCCS ISMETT (Mediterranean Institute for Transplantation and Advanced Specialized Therapies), 90127 Palermo, Italy; 13 Cardiac Surgery and Transplantology Department, Poznan University of Medical Sciences, 61-848 Poznan, Poland

**Keywords:** coronary artery disease, atherosclerosis, thrombosis, inflammation, single-cell analysis, transcriptome mapping

## Abstract

Coronary artery disease (CAD) is increasingly recognized as a thromboinflammatory disorder in which innate immune activation and coagulation are tightly coupled within the plaque microenvironment. Emerging single-cell and spatial technologies have refined this paradigm by demonstrating that these processes are not diffusely distributed but instead concentrated within discrete cellular niches. This narrative review critically evaluates mechanistic and translational studies integrating single-cell RNA sequencing, spatial transcriptomics, and ligand–receptor modeling to characterize cell–cell communication networks driving immunothrombosis in CAD. Converging evidence from single-cell and spatial studies indicates substantial heterogeneity among macrophages, neutrophils, and smooth muscle cells, with functionally distinct subpopulations contributing differentially to inflammation, matrix remodeling, and thrombogenicity. Spatial analyses further demonstrate that procoagulant and inflammatory programs converge in anatomically defined high-risk regions, particularly at the plaque shoulder and sites of endothelial dysfunction. However, whether these transcriptional states represent causal drivers or epiphenomena remains unresolved. Many insights are derived from murine models or dissociated tissues, raising concerns regarding translational relevance and loss of spatial context. Additionally, computational inference of intercellular communication remains indirect and requires functional validation. In conclusion, immunothrombosis in CAD should be interpreted as an emergent property of spatially organized cellular networks rather than a uniform inflammatory state. While these approaches identify candidate therapeutic nodes, their clinical translation and the central challenge is to distinguish causal regulatory nodes from transcriptional correlates generated by high-dimensional profiling.

## 1. Introduction

Coronary artery disease (CAD) is a chronic disease of the vascular wall with a complex etiopathogenesis, in which immunoinflammatory processes and local thrombogenicity form a coupled pathophysiological system determining plaque progression and the risk of acute coronary syndromes [1,2,3]. The contemporary approach to pathogenesis is moving away from the model reducing CAD to the consequences of lipid accumulation, as increasing evidence indicates that the key factor in lesion instability is dynamic intercellular communication between immune cells, endothelium, smooth muscle cells, and platelets, which initiates and sustains local pro-inflammatory and procoagulant programs [2,3,4,5]. In this context, immunothrombosis can be defined as an integrative mechanism linking the innate immune response with the coagulation cascade, encompassing the expression of tissue factor by myeloid cells, the formation of neutrophil extracellular traps (NETs), and the activation of platelets that perform immunoregulatory functions [6,7,8,9,10].

A significant methodological breakthrough that has enabled a deeper mechanistic understanding of CAD is the introduction of single-cell and spatial technologies. Single-cell ribonucleic acid (RNA) sequencing has revealed multidimensional heterogeneity of cell populations in atherosclerotic plaques, while spatial transcriptomics and spatial profiling have demonstrated that inflammatory and procoagulant signaling is concentrated in discrete functional microregions rather than dispersed throughout the lesion [11,12,13,14]. Additionally, modeling cellular communication via ligand–receptor pairs enables the reconstruction of signaling circuits in situ and the prioritization of regulatory axes based on network centrality [15].

For clarity, the term immunothrombosis is used throughout this manuscript to describe the coordinated activation of innate immune pathways and coagulation mechanisms as an integrated host-defense process, distinct from broader concepts such as thromboinflammation.

**Figure 1:**
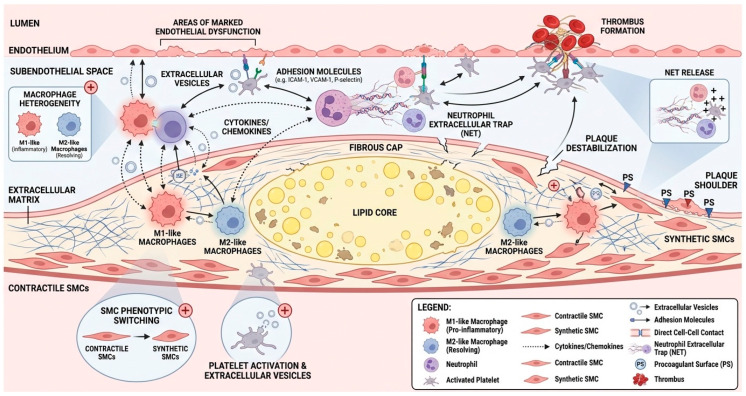
Framework synthesis illustrating immunothrombosis as a spatially organized network of interacting cellular states in coronary atherosclerosis. The schematic summarizes the conceptual organization of immunothrombotic processes within the atherosclerotic plaque. Endothelial dysfunction initiates leukocyte recruitment and promotes activation of macrophages, neutrophils, platelets, and vascular smooth muscle cells (SMCs). These cellular populations engage in bidirectional communication through cytokines, chemokines, extracellular vesicles, adhesion molecules, and direct cell–cell interactions, generating a self-reinforcing thromboinflammatory network. Macrophage heterogeneity, neutrophil extracellular trap (NET)-associated programs, SMC phenotypic switching, platelet activation, and platelet-derived extracellular vesicles contribute to local amplification of inflammation and coagulation. Spatial organization of these interactions within plaque niches, including the plaque shoulder, fibrous cap, lipid core, and regions of endothelial dysfunction, promotes extracellular matrix remodeling, expansion of procoagulant surfaces, plaque destabilization, and thrombus formation. The figure is intended as a conceptual synthesis integrating evidence from single-cell transcriptomics, spatial profiling, and mechanistic studies discussed throughout the review and does not represent direct experimental data. Created In FigureLabs. Urbanowicz, T. (2026) (ID: FL-PUB-20260617-7U24F7).

The aim of this review is to synthesize immunothrombosis in CAD as a phenomenon resulting from spatially ordered cellular communication networks, with particular emphasis on macrophage heterogeneity, NET (neutrophil extracellular traps) -associated neutrophil programs, smooth muscle cell (SMC) plasticity, the role of platelet-derived extracellular vesicles (pEVs), and the influence of clonal hematopoiesis of indeterminate potential (CHIP) on plaque thrombosis [7,11,13,16,17,18,19,20,21,22,23,24]. The conceptual framework is summarized in Figure 1.

Despite the rapid expansion of single-cell and spatial datasets, the field remains largely descriptive, with limited ability to distinguish causal regulatory architecture from transcriptional correlates. This review focuses on the mechanistic interpretation of cell–cell communication in CAD.

## 2. Literature Search Strategy

This manuscript is a narrative review that synthesizes mechanistic and translational evidence on immunothrombosis in CAD, in the context of single-cell and spatial technologies. A narrative approach was selected due to the field’s conceptual heterogeneity and the need to integrate diverse methodological frameworks, including transcriptomics, spatial profiling, and computational modeling.

Literature was identified through PubMed, Scopus, and Web of Science using combinations of terms including “atherosclerosis”, “coronary artery disease”, “immunothrombosis”, “single-cell RNA sequencing”, “spatial transcriptomics”, and “cell–cell communication”. Priority was given to primary studies employing single-cell, spatial, or multi-omics methodologies published in the last decade, supplemented by seminal mechanistic studies.

We acknowledge that narrative reviews are inherently susceptible to selection bias and do not provide the systematic reproducibility of formal systematic reviews or meta-analyses. To mitigate this limitation, priority was given to primary research studies employing single-cell RNA sequencing, spatial transcriptomics, or integrative multi-omics approaches, supplemented by seminal mechanistic studies where necessary for context.

## 3. Mechanistic Insights and Emerging Concepts

Immunothrombosis in CAD is a mechanism by which inflammatory signals increase the thrombogenicity of the lesion through coordinated endothelial activation, recruitment and reprogramming of immune cells, and modulation of the pro- and anticoagulant balance in the plaque microenvironment [2,3,5]. Endothelial activation promotes the expression of adhesion molecules, increases barrier permeability, and leads to loss of the antithrombotic phenotype, facilitating leukocyte adhesion and creating conditions for coagulation initiation at sites of erosion or rupture [5,25]. Concomitantly, proinflammatory cytokines, particularly interleukin 1β (IL-1β), interleukin 6 (IL-6), and tumor necrosis factor alpha (TNF-α), enhance tissue factor expression in myeloid cells and promote extracellular matrix degradation, thereby increasing the susceptibility of the fibrous cap to destabilization [5,26]. In high-risk regions, neutrophils and platelets form a functional thromboinflammatory module, in which NETs serve as a procoagulant scaffold, and platelet-leukocyte interactions amplify the positive feedback between inflammation and coagulation [8,9,10,27]. The most important components of the immunothrombotic axis, their biological effects on plaque, and clinical consequences are presented in Table 1, which organizes the processes from endothelial activation and tissue factor expression to NET-mediated amplification of thrombogenicity.

## 4. Single-Cell and Spatial Technologies: What They Add to Plaque Biology

Single-cell technologies have redefined plaque biology by resolving transcriptional heterogeneity; however, their interpretive power is constrained by the dissociation-induced loss of spatial context and the algorithmic imposition of discrete clusters on biologically continuous phenotypes. As a result, a central unresolved issue is whether reported ‘cell subsets’ represent stable functional entities or transient activation states [11,29,30]. In practice, this allows not only the precise distinction of cell subpopulations with distinct effector functions but also the tracking of differentiation trajectories and phenotypic reprogramming that occur in response to a changing plaque microenvironment. This is particularly important in CAD, as cells within the lesion are not static and exhibit plasticity in response to metabolic, inflammatory, and mechanical stimuli, leading to the development of intermediate phenotypes and adaptive states with significant consequences for plaque stability [29,30,31,32]. Single-cell meta-analyses and integrative cross-cohort analyses further enhance biological inference by reducing the impact of technical variability and enabling the identification of cellular states common to different patient populations and disease stages [29]. Furthermore, spatial techniques enable the assignment of inflammatory and procoagulant programs to specific tissue niches, thereby significantly increasing the interpretive resolution of destabilization mechanisms. These data indicate that thromboinflammatory activity is concentrated in discrete functional microregions, and their location relative to the endothelium, fibrous cap, and shoulder regions may determine plaque susceptibility to erosion or rupture and subsequent thrombogenesis [13,14]. Consequently, spatial analysis shifts the focus from averaged systemic markers to the local cellular architecture of signaling, enabling a more mechanistic interpretation of clinical risk. An important complement to these approaches are methods for inferring cellular communication based on ligand–receptor pairs, which reconstruct the signaling directions between cell populations and allow for prioritization of axes with high network centrality. Such modeling may help prioritize signaling pathways for future experimental validation [15]. A summary of the main technological approaches, their mechanistic value, and common limitations is presented in Table 2 to facilitate the interpretation of how single-cell and spatial data support inferences about immune-thrombotic networks.

### 4.1. Single-Cell and Spacial Technologies: Human vs Mouse Studies

A critical limitation across many studies is the reliance on murine models, which may not fully recapitulate human plaque biology [35,36]. While single-cell analyses in mice provide mechanistic insight, species-specific differences in immune composition and vascular biology raise concerns regarding direct translational relevance. Integrating human spatial datasets with experimental models remains essential to resolve this gap.

### 4.2. Single-Cell Limitations

While single-cell RNA sequencing has transformed the understanding of cellular heterogeneity, several limitations must be considered when interpreting these data [37]. The dissociation process inherently disrupts tissue architecture, eliminating spatial context and potentially introducing transcriptional artifacts [38]. Furthermore, clustering algorithms rely on computational assumptions that may artificially define discrete populations within what is biologically a continuum. Single-cell data have fundamentally reshaped the understanding of cellular heterogeneity in atherosclerosis. However, whether the identified transcriptional states represent stable functional entities or transient activation programs remains unresolved. In particular, the increasing granularity of clustering risks overinterpreting biologically continuous processes as discrete cell populations, raising concerns that some “novel subsets” may reflect analytical findings rather than physiologically distinct states.

### 4.3. Spatial Transcriptomics Limitations

Spatial transcriptomic approaches partially address these limitations by preserving tissue context; however, current platforms often lack single-cell resolution, making it difficult to confidently assign gene expression to specific cell types [39]. Additionally, spatial data are highly dependent on tissue quality and sampling strategy and may not fully capture dynamic cellular interactions over time.

## 5. Macrophage Heterogeneity and Thromboinflammatory Phenotypes

Macrophages are central regulators of plaque inflammation [33,40]. A critical unresolved question is whether foam cell identity is best defined by lineage or function. While murine lineage-tracing suggests substantial SMC contribution, human single-cell data rely on marker inference, raising the possibility that current models both overestimate lineage specificity and underestimate functional convergence.

High-resolution single-cell data and contemporary reviews consistently indicate that the dichotomous M1/M2 paradigm has limited explanatory value, as the actual functional states of macrophages form a continuum encompassing both lipid adaptation phenotypes and resolution-related programs, as well as proinflammatory, tissue-remodeling, and prodegradative phenotypes, the participation and significance of which depend on the microenvironment and the stage of disease progression [33,40,41]. From the perspective of thromboinflammation, it is particularly important that individual macrophage subtypes differ in their ability to maintain cytokine networks, modulate efferocytosis, influence extracellular matrix remodeling, and interact with other effector cells, including neutrophils and platelets. Among the best-characterized populations are TREM2+ (triggering receptor expressed on myeloid cells 2) macrophages, which exhibit a lipid-adaptive profile and express genes supporting lipid metabolism, lysosomal function, and survival in a lipid-rich environment. This favors the foam cell phenotype and may be associated with a more homeostatic, stabilizing component of the response in advanced lesions [41,42]. A distinct, clearly pathogenic nature is attributed to the SPP1+ (secreted phosphoprotein 1) macrophage population, defined by osteopontin expression, which correlates with the development of a necrotic core, increased cellular stress, angiogenesis, and features of plaque instability, including processes promoting intraplaque hemorrhage and structural weakening [43]. In parallel, subpopulations with a strong pro-inflammatory signature are present, maintaining active cytokine and chemokine networks and may act as “amplifiers” for the recruitment of additional immune cells, including monocytes, thereby perpetuating inflammation and structural destabilization through tissue remodeling and matrix degradation [36]. A comprehensive overview of the most important macrophage subpopulations, their signatures, and functional consequences in CAD is presented in Table 3, which summarizes the relationship between macrophage status and thromboinflammatory and destabilizing potential.

### Macrophage Controversy

A central unresolved issue concerns the origin and functional identity of foam cells. While lineage-tracing studies in murine models suggest a substantial contribution of smooth muscle cell–derived populations, single-cell transcriptomic analyses in human plaques often rely on marker-based inference, which may not reliably distinguish lineage [44,45]. This discrepancy raises the possibility that current models overestimate the contribution of SMC-derived foam cells or, conversely, underestimate their functional plasticity. Resolving this issue is critical, as it directly influences therapeutic strategies targeting either inflammatory recruitment or vascular cell reprogramming.

## 6. Neutrophils and NETosis Programs in CAD

Neutrophils are currently recognized as important mediators of thromboinflammatory disease in coronary artery disease, particularly in the context of plaque destabilization and the pathogenesis of acute events, which involve a rapid increase in proinflammatory and procoagulant processes within the lesion [8,27]. Current data, including high-resolution analyses, indicate that neutrophils present in atherosclerotic lesions are not a homogeneous population but can assume heterogeneous functional states dependent on the microenvironment. This includes, among others, subpopulations predisposed to NETosis, whose pathophysiological significance may exceed the mere number of neutrophils in the circulation or tissue [27]. This approach shifts the interpretation from quantitative parameters towards a qualitative assessment of neutrophil activation states and their interactions with other components of the thromboinflammatory network. NETs constitute a mechanism that integrates innate immunity with coagulation by providing a spatial chromatin matrix rich in histones and granular proteins with procoagulant potential. NETs can directly damage the endothelium, increase the exposure of prothrombotic reaction surfaces, and create a scaffold that promotes local thrombin generation and thrombus stabilization [28,46]. As a result, NETs function as effectors that simultaneously amplify the local inflammatory response and increase thrombogenicity in high-risk niches, particularly near dysfunctional endothelium.

Single-cell analyses also suggest the existence of programs termed “NET-cycling,” in which increased expression of Toll-like receptors (TLRs) and chemokine axes perpetuates loops of myeloid cell recruitment and activation. In this model, NETosis is not an isolated event but rather part of a self-perpetuating circuit in which mediators released during neutrophil activation promote further recruitment and activation of effector cells, enhancing local thrombogenicity and sustaining thromboinflammation [27]. Neutrophil-platelet interactions are also a crucial component of this circuit, which may increase susceptibility to NETosis and simultaneously exacerbate the procoagulant consequences of NETs by enhancing platelet activation and coagulation.

The most important feedback axes involving NETs and their network consequences are presented in Table 4, which highlights the links among NETosis, platelet activation, thrombin generation, and modulation of macrophage phenotype.

Importantly, the causal role of NETs in plaque destabilization remains debated. While experimental models demonstrate prothrombotic effects, human data are largely associative, and NET markers may reflect downstream consequences of inflammation rather than primary drivers. This distinction is crucial when considering NET-targeted therapies, which may fail if NETosis represents a secondary amplification loop rather than an initiating mechanism. While NETosis has been extensively characterized in bulk and experimental systems, recent single-cell analyses extend this framework by identifying transcriptionally distinct neutrophil subsets within plaques, including interferon-responsive and oxidative stress-associated populations, suggesting functional specialization beyond classical NET formation.

Recent single-cell studies extend classical NETosis models by identifying transcriptionally distinct neutrophil subsets with interferon-responsive and oxidative stress-associated programs within plaques. Spatial mapping further indicates that these subsets localize to vulnerable plaque regions, suggesting that neutrophil function is spatially constrained rather than systemically uniform.

## 7. Smooth Muscle Cell Plasticity and Immune-like Switching

Smooth muscle cells (SMCs) in coronary artery disease exhibit significant phenotypic plasticity, and their reprogramming is increasingly recognized as a key mechanism linking vessel wall remodeling with local inflammation and increasing plaque thrombogenicity [31,47]. From a pathogenetic perspective, SMCs play a role beyond structure; they also help shape the lesion microenvironment through phenotypic transitions induced by lipid overload, inflammatory stimuli, and mechanical signals. The importance of this population is reinforced by genetic data indicating that CAD-associated variants modulate the expression of SMC-specific genes, suggesting that deregulation of SMC programs may be a significant component of disease susceptibility [47]. Single-cell and spatial profiling have demonstrated that SMCs can suppress the contractile program and enter transitional states with immunological features that partially overlap with the signatures of macrophages, fibroblasts, and antigen-presenting cells [31]. This phenomenon is of significant interpretive importance, as it indicates that some of the “immune signatures” detected within the plaque may result not only from hematopoietic cell infiltration but also from reprogramming of vessel wall cells. Mechanistically, these transitions are driven by transcriptional regulators and chromatin remodeling, leading to the silencing of contractile genes and the activation of programs in lipid metabolism, lipoprotein uptake, and cytokine signaling [48]. Consequently, SMCs may acquire the ability to actively participate in local inflammatory cross-talk, including by modulating immune cell recruitment and tissue response to damage. From a functional perspective, the phenotypes of SMC-derived foam cells are particularly important, as they may exhibit impaired efferocytosis and cholesterol efflux, which promotes lipid accumulation, increased cellular stress, and enlargement of the necrotic core [48,49]. Moreover, their preferential localization in the fibrous cap and shoulder regions affects the structural integrity of the plaque by modulating extracellular matrix remodeling and degradation, as well as by interacting with immune cells and the endothelium in regions particularly susceptible to destabilization [50]. Collected data suggest that therapies that globally suppress inflammation may not eliminate the maladaptive plasticity of SMCs, whereas more selective modulation of the SMC phenotypic trajectory may represent a promising avenue of intervention for plaque stabilization, with potentially lower risk of systemic effects [51]. These findings suggest that SMC plasticity (Table 5) is not merely a secondary response but may represent a parallel axis of disease progression that is insufficiently addressed by current anti-inflammatory strategies.

## 8. Spatial Microenvironments and High-Risk Plaque Niches

Immunothrombosis in CAD is not a homogeneous process or evenly distributed throughout the lesion, but rather a phenomenon strongly dependent on the spatial organization of the plaque microenvironment, including mediator gradients, local differences in the composition of the extracellular matrix, and the action of hemodynamic and biomechanical forces at the vessel lumen–wall interface [34,56]. Consequently, the same cell type may exhibit distinct functional programs depending on its niche, and key inflammatory and procoagulant axes may undergo focal amplification in zones particularly susceptible to damage. Advances in spatial transcriptomics and transcriptional mapping have demonstrated that the risk of plaque destabilization is associated with the colocalization of specific cellular states and the activation of selected signaling axes in areas of increased structural vulnerability, such as shoulder regions and areas of endothelial dysfunction [13,14]. Spatial data suggest that these zones are where signals for leukocyte recruitment, proinflammatory activation, and coagulation programs converge, promoting the transition from chronic inflammation to thrombotic events. High-resolution spatial profiling in atherosclerosis models and analyses in human tissues support the concept of pathogenic hotspots, where metalloproteinase induction, enhanced matrix degradation, and activation of procoagulant programs occur, directly translating into weakened cap integrity and increased thrombogenicity [26,57]. This process explains why systemic indicators do not always reflect local risk of destabilization, as critical mechanisms may be confined to narrow, highly active microregions. In this approach, intercellular communication also encompasses non-classical pathways, including extracellular vesicles and RNA regulators, which enable signal transfer between different plaque compartments and the circulation and may thus amplify thromboinflammatory feedback loops in ways that are difficult to capture with analyses based solely on soluble mediators [58,59]. These mechanisms promote both the perpetuation of local inflammatory networks and the modulation of the phenotype of vascular and myeloid cells in high-risk zones.

**Figure 2:**
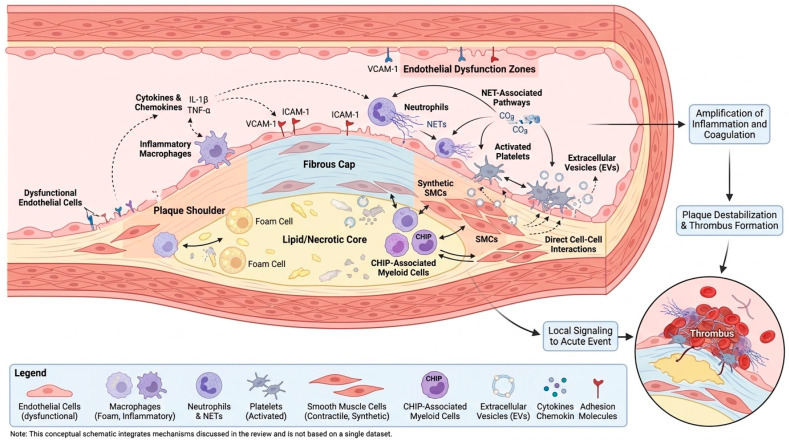
Selected mechanistic pathways within the spatial immunothrombotic network in coronary atherosclerosis. The figure illustrates key cellular and molecular interactions linking endothelial dysfunction, macrophages, neutrophils, platelets, smooth muscle cells, extracellular vesicles, and CHIP-associated myeloid cells. These interconnected signaling pathways amplify local inflammation and coagulation within high-risk plaque niches, promoting plaque destabilization, thrombosis, and acute coronary events. The schematic represents a conceptual synthesis of mechanisms discussed in the review and is not based on a single experimental dataset. Dashed arrows indicate indirect regulatory interactions, chemotactic signaling, or conceptual pathways supported by cumulative evidence, whereas solid arrows represent direct biological processes, cellular migration, or experimentally established mechanistic interactions. Created in FigureLabs by Urbanowicz, T (2026) (ID: FL-PUB-20260617-56CRRM).

The most important examples of vulnerable plaque regions, their dominant cell populations, and associated functional processes are presented in Figure 2 and Table 6, which synthesizes a spatial interpretation useful for analyzing the mechanisms of destabilization.

A key limitation in interpreting SMC plasticity arises from the reliance on transcriptomic signatures without definitive lineage tracing in human tissue. As a result, the extent to which SMC-derived cells functionally mimic macrophages versus represent distinct pathological entities remains unresolved. This ambiguity complicates efforts to therapeutically target these populations, as interventions aimed at reducing inflammation may not adequately address maladaptive vascular cell reprogramming.

## 9. Extracellular Vesicles and Platelet-Derived Signaling

Extracellular vesicles (EVs) constitute an essential, multilayered mechanism of intercellular communication, enabling the transfer of proteins, lipids, and nucleic acids between cells in a context-dependent manner, including atherosclerosis patophysiology [60,61,62]. Platelet extracellular vesicles (pEVs) are particularly important in the cell-to-cell crosstalk; they are among the most abundant EV fractions and can act as active vectors of thromboinflammatory signaling, linking hemostasis with the innate immune response [16,17,18,19,20].

An important limitation is that EV biology has been largely studied in circulation, whereas emerging spatial data suggest that vesicle-mediated signaling is highly localized. Integrating EV biology with spatial transcriptomics may therefore redefine EVs not as systemic mediators but as niche-specific amplifiers of thromboinflammatory signaling.

pEVs are released in response to hemodynamic stimuli, oxidative stress, and inflammatory signals, so that their abundance and composition may reflect the current state of platelet and vascular activation while also modifying local processes within the plaque [19,20]. The pEV cargo includes lipids with procoagulant potential, adhesion proteins, and inflammatory mediators, as well as regulatory RNAs that can reprogram the phenotype of target cells, including endothelium, monocytes, and smooth muscle cells, enhancing the inflammation-coagulation interface within the lesion [19,20,63,64,65]. One of the key procoagulant mechanisms of pEVs is the exposure of phosphatidylserine on their surface, which increases the catalytic surface area for the coagulation reaction and enhances thrombin generation, particularly at sites of plaque erosion or rupture, where the integrity of the endothelial barrier is compromised [61,62]. This mechanism is important not only during acute events but also in the chronic perpetuation of local thrombogenicity in spatial microenvironments. Regardless of their direct role in thrombin generation, pEVs exhibit immunomodulatory properties, as they can induce the expression of the adhesion molecules ICAM-1 and VCAM-1 and support proinflammatory programs in endothelial cells, thereby increasing leukocyte adhesion and intimal infiltration, thereby promoting the maintenance of an active inflammatory microenvironment [16,18,20,62]. Furthermore, the transport of microRNAs, including those associated with the regulation of the inflammatory response and metabolism, suggests the possibility of a long-term effect of pEVs on the transcriptional programs of vascular and myeloid cells, consistent with the epigenetic-transcriptional model of the perpetuation of proatherogenic phenotypes [63,64,65]. The platelet-neutrophil axis is particularly important in the pathogenesis of thromboinflammation, where pEVs may act as stimuli that initiate or exacerbate NETosis. The produced NETs, in turn, enhance platelet activation and coagulation, creating a self-perpetuating prothrombotic system that couples the innate immune response with hemostasis and increases the likelihood of an acute event within the unstable lesion [66,67,68]. The clinical importance of EVs and pEVs is supported by observations indicating their association with microcirculatory dysfunction and the risk of cardiovascular events, as well as data suggesting that metabolic disorders, including hyperglycemia, can modify both the abundance and load of pEVs, making them potential indicators of the severity of atherothrombotic processes [62,69,70,71].

A key unresolved question is how EV-mediated signaling operates within the spatial architecture of the plaque. While most EV studies are based on circulating vesicles, emerging spatial transcriptomic data suggest that EV signaling may be highly localized and niche specific. Integrating EV biology with spatial mapping could redefine vesicles as focal amplifiers of thromboinflammatory signaling rather than systemic mediators. At the same time, despite their pathogenic role, pEVs are considered potential therapeutic platforms capable of selectively delivering inflammatory inhibitors to vascular niches, which is consistent with the concept of spatially targeted interventions targeting the lesion microenvironment [72,73,74]. The main mechanisms of action of pEV and their translational implications are presented in Table 7, which includes both procoagulant, immunomodulatory, and biomarker aspects.

A major limitation in EV research is the difficulty in assigning vesicle origin and target specificity in vivo, particularly within complex tissue environments such as atherosclerotic plaques. As a result, current models of EV-mediated communication remain largely inferential and may overestimate their functional specificity.

## 10. CHIP as a Systemic Modifier of Plaque Thromboinflammation

Clonal hematopoiesis of indeterminate potential (CHIP) describes the age-dependent expansion of clones of hematopoietic stem and progenitor cells harboring somatic mutations, most often in epigenetic and signaling regulatory genes such as DNMT3A, TET2, ASXL1, or JAK2, in the absence of overt hematological disease [22]. This phenomenon is of particular importance to the pathophysiology of CAD because it provides a model in which the hematopoietic system can program the properties of the innate immune effector cells systemically before they reach the vessel wall, thereby modulating the thromboinflammatory architecture of the plaque. Epidemiological and clinical data indicate that CHIP is associated with an increased risk of cardiovascular events, and this association is also observed in early and non-obstructive forms, suggesting that CHIP may participate not only in the progression but also in the initiation of vascular dysfunction [23,24,74]. Mechanistically, CHIP mutations may reprogram monocyte and macrophage function by enhancing NLRP3 inflammasome activity and increasing IL-1β expression, which promotes endothelial activation, increased adhesion molecule expression, and leukocyte recruitment to the vessel wall [75]. In this way, CHIP may exacerbate chronic, low-grade systemic inflammation, thereby increasing susceptibility to the development and perpetuation of proinflammatory niches within the plaque. In the case of JAK2 mutations, increased signaling and a greater propensity for thrombogenesis have also been reported, supporting the concept that, in selected patients, CHIP may constitute a systemic prothrombotic component contributing to the risk of acute events [75,76]. Furthermore, epigenomic studies indicate that CHIP correlates with distinct DNA methylation patterns that may modulate the expression of genes involved in the inflammatory response and vascular function, creating a mechanistic bridge between clonal expansion and the perpetuation of a proinflammatory phenotype in the myeloid lineage [77]. The cardiovascular risk associated with CHIP is not uniform and depends on both clone size and mutation type. This suggests the possibility of clinically useful patient stratification, in which the presence of specific mutations or higher clonal contribution identifies subgroups at higher risk of events and potentially greater susceptibility to anti-inflammatory interventions targeting the IL-1β/IL-6 axis [78,79,80]. In this context, CHIP may represent one biological explanation for residual risk that persists despite optimal control of classic risk factors, while also pointing the way toward personalizing therapy based on immunological mechanisms. The mechanisms linking CHIP to thrombosis in CAD, along with their clinical consequences, are summarized in Table 8.

However, the causal contribution of CHIP to cardiovascular events remains incompletely resolved, as CHIP may act as a marker of biological aging rather than an independent driver in all patient subsets. Disentangling these effects is essential for determining whether CHIP represents a therapeutic target or primarily a risk stratification tool.

## 11. Translational Outlook: Therapeutic Nodes and Precision/Spatial Delivery

From a systems biology perspective, CAD progression and instability may result from the activity of a limited number of intercellular communication nodes that integrate metabolic, inflammatory, and biomechanical signals within the plaque. These nodes are high-network centrality elements, as they connect multiple parallel effector pathways, and their modulation can induce disproportionately large changes in the dynamics of the entire thromboinflammatory system [81,82,83]. Axes of particularly high network importance include the IL-1β/IL-6–STAT3 pathway, NLRP3 inflammasome activation, and chemokine networks that determine the recruitment, retention, and functional reprogramming of leukocytes in the lesion microenvironment [81,83,84,85]. In this context, interventions targeting nodes that amplify inflammatory circuits may be more effective than strategies that block individual mediators, as they act on the network architecture rather than isolated segments of signaling pathways [81,86]. In parallel, therapeutic approaches targeting the immunothrombotic interface that limit pathological thrombogenesis with minimal impact on physiological hemostasis are gaining importance. This particularly concerns the P-selectin/PSGL-1 axis, which is responsible for the formation of platelet-leukocyte aggregates and enhances myeloid cell recruitment, as well as PAD4-dependent NETosis mechanisms, which provide a procoagulant scaffold and enhance thrombin generation in spatial microenvironments [28,87]. Such targeting is consistent with the concept of decoupling thrombinitis from hemostasis, which aims to disrupt the feedback loops that drive thrombosis without significantly impairing the physiological ability to control bleeding. Because numerous spatial studies indicate that pathogenic processes in CAD are concentrated in vulnerable plaque regions, spatially controlled drug delivery strategies are being developed to modulate pathogenic states in situ. This includes nanocarriers that utilize microenvironmental signatures, such as altered pH, redox state, and overexpression of adhesion molecules, which are expected to promote selective drug accumulation in areas of highest inflammatory and procoagulant activity and limit adverse effects resulting from systemic exposure [88,89,90,91]. Complementarily, integrating omics data, including spatial omics, with computational modeling can support predictions of drug retention and distribution in heterogeneous lesions and optimize the selection of therapeutic targets by identifying dominant pathways in specific plaque phenotypes [92,93]. This approach creates a framework for precision therapy, in which therapeutic decisions are linked to the lesion’s molecular and cellular architecture rather than solely to average clinical parameters.

A summary of key therapeutic nodes and their targeting logic in the context of immunothrombosis is presented in Table 9, which includes both immunological targets and targets at the immunothrombotic interface, as well as spatial targeting strategies.

The current body of evidence suggests that immunothrombosis in CAD is not driven by isolated cellular actors but by emergent properties of interacting networks. However, the field remains limited by methodological fragmentation, with single-cell, spatial, and functional studies often performed in isolation. A major future challenge lies in integrating these approaches to distinguish causative mechanisms from epiphenomena and to identify actionable therapeutic targets with true clinical relevance.

The central translational challenge is not identifying additional molecular pathways but prioritizing regulatory nodes whose perturbation produces system-level effects. Without such prioritization, therapeutic strategies risk targeting peripheral components of the network while leaving core drivers intact. A major limitation of current therapeutic strategies is their focus on individual mediators rather than network architecture. Targeting high-centrality nodes may achieve system-level modulation, whereas peripheral inhibition is unlikely to alter disease trajectory. Future progress will depend on integrating spatial, functional, and longitudinal data to distinguish causal architecture from descriptive complexity.

## 12. From Description to Causal Inference: Interpreting Single-Cell and Spatial Data in Immunothrombosis

The rapid adoption of single-cell and spatial technologies has fundamentally reshaped the understanding of coronary artery disease (CAD), enabling unprecedented resolution of cellular heterogeneity and tissue organization [94,95,96]. However, a critical conceptual limitation remains: these approaches are inherently descriptive and do not, in isolation, establish causality. As a result, the field faces a growing risk of conflating high-dimensional molecular characterization with mechanistic understanding.

Single-cell RNA sequencing (scRNA-seq) and related approaches identify transcriptional states, infer cellular trajectories, and reconstruct intercellular communication networks based on ligand–receptor co-expression. While these analyses provide powerful hypotheses regarding cellular interactions and functional specialization, they do not directly demonstrate that a given cell state drives disease progression or plaque destabilization. Instead, they capture snapshots of gene expression that may reflect downstream consequences of upstream processes, adaptive responses to microenvironmental stress, or transient activation states without independent pathogenic significance. This distinction is particularly important in atherosclerosis, where inflammation, lipid metabolism, and tissue remodeling are tightly intertwined and dynamically regulated.

A central challenge, therefore, lies in distinguishing causal regulatory nodes from correlative transcriptional signatures. For example, macrophage subpopulations defined by inflammatory or lipid-associated gene expression may be interpreted as drivers of plaque instability; however, without functional perturbation, it remains unclear whether these states actively promote disease or merely mark regions of advanced pathology [97]. Similarly, neutrophil programs associated with NETosis may reflect amplification loops rather than initiating events, and smooth muscle cell (SMC) phenotypic transitions may represent adaptive remodeling responses rather than primary pathogenic triggers [98].

This limitation is further compounded by the increasing granularity of clustering approaches. As analytical resolution improves, there is a tendency to define progressively smaller “cell subsets,” raising the possibility that some reported populations reflect computational partitioning of continuous biological processes rather than discrete functional entities. Consequently, the identification of transcriptionally distinct clusters should not be equated with the discovery of biologically independent cell types.

To address these challenges, there is a growing need to integrate perturbation-based and spatially resolved functional validation into the study of immunothrombosis. Perturbation models—such as genetic manipulation, targeted inhibition of signaling pathways, or controlled modulation of specific cell populations—are essential to establish whether candidate pathways identified by single-cell analyses exert causal effects on plaque biology. In parallel, spatial-functional approaches, including in situ protein mapping, imaging-based transcriptomics, and lineage tracing (where feasible), are required to confirm that inferred interactions occur within relevant anatomical niches and contribute to local thromboinflammatory activity.

Within this framework, it is useful to distinguish between transcriptional centrality and regulatory centrality. Single-cell network analyses often prioritize pathways or cell populations based on measures such as connectivity, differential expression, or inferred ligand–receptor interactions. While these metrics identify elements that are prominent within transcriptional networks, they do not necessarily correspond to nodes that exert dominant control over system behavior. In other words, a pathway that appears highly connected at the transcriptomic level may not be functionally indispensable, whereas less prominent nodes may exert disproportionate regulatory influence through non-linear or context-dependent effects. This distinction has direct translational implications, as therapeutic strategies targeting transcriptionally prominent but functionally peripheral pathways are unlikely to achieve meaningful clinical impact.

The integration of single-cell, spatial, and perturbational data, therefore, represents a critical next step in the field. Rather than expanding catalogues of cellular states, future studies should aim to construct causal hierarchies of thromboinflammatory regulation, identifying which interactions are necessary and sufficient to drive plaque destabilization. This shift from descriptive complexity to mechanistic prioritization is essential for translating systems-level insights into effective therapeutic strategies.

Ultimately, the value of single-cell and spatial technologies will depend not on their ability to generate increasingly detailed molecular maps, but on their integration with functional frameworks that can distinguish drivers from bystanders. Without this transition, there is a risk that the field will accumulate descriptive knowledge without achieving corresponding advances in causal understanding or clinical intervention.

### Key Unresolved Questions

Despite rapid technological progress, several fundamental questions remain unresolved:

1. Which transcriptional states represent causal drivers versus adaptive responses?
2. To what extent do murine models recapitulate human plaque biology?
3. Are spatially defined niches stable entities or dynamic states?
4. Which pathways are both biologically central and therapeutically tractable?

Addressing these questions will determine whether systems-level insights translate into clinically meaningful interventions.

## 13. Conclusions and Future Directions

Immunothrombosis in coronary artery disease should be interpreted as the result of spatially organized cellular communication networks, in which inflammatory and procoagulant processes mutually reinforce each other, creating conditions for plaque destabilization and acute thrombotic events. Data from single-cell and spatial technologies indicate that key pathogenic mechanisms are concentrated in specialized functional niches, and clinical risk stems not only from the presence of specific cells but also from their transcriptional states, spatial proximity, and signaling network topology. Macrophage heterogeneity, NET-associated neutrophil programs, smooth muscle cell plasticity, and platelet and extracellular vesicle signaling comprise the core of thrombotic–inflammatory plaque biology and offer potential therapeutic targets. In parallel, clonal hematopoiesis represents a significant systemic modifier of the proinflammatory and prothrombotic phenotype, supporting the concept of patient stratification and more precise therapies. The key translational challenge is not identifying additional molecular components, but prioritizing pathways whose modulation produces meaningful changes in plaque biology without disrupting systemic immune and hemostatic balance. Future studies should integrate spatial mapping with functional validation to distinguish pathogenic nodes from adaptive mechanisms and design interventions that selectively disrupt critical network connections in vulnerable plaque regions while minimizing disruption of systemic immunity and physiological hemostasis.

## Figures and Tables

**Table 1:** Determinants of the thromboinflammatory axis in coronary artery disease and their clinical significance.

Immunothrombotic Axis Component	Mechanism	Biological Effect Within the Plaque	Clinical Implication	References
Endothelial activation	Endothelial activation induces a pro-inflammatory program and upregulates adhesion molecules, which facilitate leukocyte adhesion and transendothelial migration.	Recruitment of immune cells increases, and features of vascular barrier dysfunction become more pronounced.	Susceptibility to endothelial erosion and the risk of local thrombogenicity increase.	[2,3,5,25]
Tissue factor expression in myeloid cells	Monocytes and macrophages can induce tissue factor expression, which initiates the coagulation cascade within the lesion microenvironment.	Enhanced thrombin generation promotes thrombus stabilization at sites of plaque injury.	The risk of thrombus formation during acute coronary syndromes increases.	[2,26]
NETs as a procoagulant scaffold	Neutrophil extracellular traps form a chromatin network enriched in histones and granular proteins, which amplifies procoagulant activity and endothelial injury.	NETs provide a scaffold for coagulation reactions and promote activation of cells and mediators at sites of plaque destabilization.	The likelihood of an acute thrombotic event and microthrombus formation increases.	[8,9,10,27,28]
Platelet–leukocyte interactions	Platelets form aggregates with leukocytes and release soluble mediators that enhance innate immune activation and promote coagulation.	A thromboinflammatory state is sustained and local recruitment loops are intensified.	The risk of cardiovascular events and progression of plaque instability increases.	[9,10,27]
Extracellular matrix proteolysis	Matrix metalloproteinases and other matrix-degrading enzymes weaken the fibrous cap, increasing its susceptibility to structural damage.	Structural integrity of the fibrous cap deteriorates.	The risk of plaque rupture and subsequent thrombosis increases.	[5,26]

Abbreviations: NET—neutrophil extracellular traps.

**Table 2:** Single-cell and three-dimensional technologies in CAD research: mechanistic value and interpretative limitations.

Research Approach	Description	Key Mechanistic Contribution to CAD	Limitation to Consider	References
scRNA-seq	Single-cell RNA sequencing characterizes the transcriptional state of individual cells present within the lesion.	This approach enables the discovery of novel subpopulations and phenotypic continuums and supports the identification of transitional cellular states.	The method does not preserve tissue topology on its own and requires integration with spatial data.	[11,29,33]
Spatial transcriptomics	Spatial transcriptomics maps gene expression across the tissue’s anatomical locations.	This approach identifies functional microregions in which inflammatory and procoagulant programs are concentrated.	Resolution depends on the platform and may limit the assignment of signals to individual cells.	[12,13,14,34]
Proteomic profiling and multiplex imaging	Multiplex imaging enables the simultaneous assessment of multiple protein markers and cellular colocalization.	This method supports functional validation and the identification of pathogenic cellular neighborhoods.	The analysis is computationally complex and sensitive to pre-analytical variability.	[14,34]
Ligand–receptor modeling	Ligand–receptor algorithms infer potential communication pathways based on gene expression patterns across cell populations.	This approach prioritizes candidate signaling pathways.	Inference is indirect and requires experimental validation.	[15]
Multi-omics integration	Multi-omics integration combines transcriptomic data with epigenomic, proteomic, or spatial information.	This approach links cellular states to regulatory programs and environment-dependent differentiation trajectories.	Technical differences between platforms require harmonization and batch-effect control.	[11,14,29]

Abbreviations: scRNA-seq—Single-cell RNA sequencing (a high-resolution transcriptomic technique used to analyze gene expression at the level of individual cells, enabling identification of cellular heterogeneity and distinct cell populations).

**Table 3:** Macrophage subpopulations in atherosclerotic plaques and their role in the thromboinflammatory biology of CAD.

Macrophage Subpopulation	Description	Consequence for the Plaque Microenvironment	Relevance to Clinical Risk	References
TREM2+ macrophages with a lipid-adaptive profile	TREM2+ macrophages express genes that support lipid metabolism and lysosomal function and may promote controlled efferocytosis.	This population may limit excessive inflammation while adapting to a lipid-rich environment.	This phenotype is often interpreted as relatively stabilizing in advanced lesions.	[41,42]
Macrophages with a pro-inflammatory signature	Pro-inflammatory macrophages maintain high expression of cytokine-related genes and innate immune response pathways without a dominant foam-cell signature.	This population acts as an amplifier of local cytokine networks and cellular recruitment.	Sustained inflammation promotes lesion progression and increases the likelihood of destabilization.	[40,41]
SPP1+ macrophages associated with osteopontin	SPP1+ macrophages are characterized by osteopontin expression and cellular stress and apoptosis signatures that correlate with necrotic core development.	This population promotes degenerative processes, angiogenesis, and intraplaque hemorrhage.	An increased contribution of this phenotype is associated with a higher risk of plaque instability.	[43]

Abbreviations: SPP1—Secreted phosphoprotein 1 (osteopontin) (a multifunctional protein involved in inflammation, tissue remodeling, and angiogenesis, often expressed by activated macrophages), TREM2—Triggering receptor expressed on myeloid cells 2 (a receptor involved in lipid sensing, phagocytosis, and regulation of macrophage inflammatory responses).

**Table 4:** Neutrophil extracellular traps as a network module of thromboinflammation in CAD.

Interaction Axis	Description	Biological Consequence	Functional Significance	References
Neutrophil–platelet interaction	Neutrophils and platelets mutually enhance their activation, increasing the propensity for NETosis and aggregation.	A positive feedback loop emerges, increasing local pro-inflammatory and procoagulant activity.	This module promotes the transition from chronic inflammation to a thrombotic event.	[10,27]
NET–coagulation interaction	NETs provide a matrix for coagulation reactions and facilitate local thrombin generation.	Enhanced thrombin generation increases thrombus stability at the site of lesion destabilization.	This mechanism reinforces thrombogenicity within high-risk plaques.	[10,28]
NET–macrophage interaction	NET components can shift macrophages toward pro-inflammatory phenotypes and activate innate immune pathways.	Local inflammation is sustained, and cytokine circuits are strengthened.	This loop promotes plaque progression and destabilization by amplifying thromboinflammation.	[27,28]

Abbreviations: NET—Neutrophil extracellular trap (a chromatin-based structure released by activated neutrophils that promotes inflammation and coagulation).

**Table 5:** Representative Single-Cell Studies Demonstrating SMC Plasticity in CAD.

Model/Material	Major Finding Related to SMC Plasticity	Clinical Relevance	References
Human and murine atherosclerotic plaques analyzed by scRNA-seq and genetic association studies	Identified phenotypically modulated SMCs regulated by TCF21 and demonstrated extensive SMC state transitions during atherosclerosis.	Suggested that SMC modulation may be protective and represents a therapeutic target distinct from inflammation.	[52]
Mouse lineage-tracing models and human scRNA-seq datasets	Identified the SEM (stem cell/endothelial cell/monocyte-like) transitional state arising from SMCs and revealed major SMC phenotypic plasticity.	Demonstrated remarkable SMC plasticity and highlighted potential therapeutic targets regulating cell-state transitions.	[32]
Human carotid atherosclerotic plaques analyzed by scRNA-seq	Human plaque scRNA-seq identified multiple SMC-like, fibromyocyte, and transitional populations, demonstrating marked cellular plasticity.	Showed that plaque SMCs cannot be adequately described by traditional marker expression alone.	[53]
ApoE−/− mouse lineage-tracing model of atherosclerosis	Lineage tracing showed that SMC-derived cells constitute the majority of foam cells in advanced murine plaques.	Supported the concept that SMCs contribute directly to plaque progression and instability.	[44]
Mouse lineage-tracing model combined with scRNA-seq	Demonstrated that SMC-derived cells can lose canonical SMC markers and acquire macrophage-like phenotypes.	Challenged the traditional view that plaque foam cells are predominantly macrophage-derived.	[54]
Mouse lineage-tracing models with single-cell transcriptomic analysis	Showed that KLF4-dependent SMC transitions generate diverse plaque-cell states including foam-cell-like populations.	Highlighted the limitations of marker-based lineage assignment in human single-cell datasets.	[55]

Abbreviations: ApoE−/−—Apolipoprotein E-deficient (a genetically modified mouse model widely used to study atherosclerosis); CAD—Coronary artery disease; KLF4—Krüppel-like factor 4 (a transcription factor regulating smooth muscle cell phenotypic switching and cellular plasticity); scRNA-seq—Single-cell RNA sequencing (a high-resolution transcriptomic technique used to analyze gene expression at the level of individual cells); SEM—Stem cell/endothelial cell/monocyte-like (a transitional smooth muscle cell-derived state identified during atherosclerosis progression); SMC—Smooth muscle cell (a vascular wall cell type involved in vessel structure, remodeling, and plaque biology); TCF21—Transcription factor 21 (a regulator of smooth muscle cell differentiation and phenotypic modulation).

**Table 6:** Vulnerable plaque regions in atherosclerotic plaques and their functional interpretation in the context of destabilization.

Anatomical-Functional Niche	Description	Dominant Biological Processes	Relevance to Thrombogenicity	References
Shoulder region	The shoulder region often contains colocalized macrophages, neutrophils, and platelet-derived elements in close proximity to dysfunctional endothelium.	Inflammatory programs, NET-dependent procoagulant activity, and myeloid cell recruitment are intensified in this region.	This niche is characterized by a high susceptibility to destabilization and thrombotic events.	[13,14,27]
Fibrous cap	The fibrous cap contains smooth muscle cells, transitional phenotypes with immune-like features, and immune cells capable of modulating the extracellular matrix.	Extracellular matrix degradation and weakening of mechanical properties occur, increasing susceptibility to structural injury.	Cap weakening increases the likelihood of plaque rupture and subsequent thrombosis.	[26,50]
Areas of neovascularization and intraplaque hemorrhage	These areas exhibit pro-angiogenic and pro-degradative phenotypes, including SPP1+ macrophage populations.	Angiogenesis, vascular leakage, and intraplaque hemorrhage develop, thereby amplifying instability.	The risk of lesion progression and acute events increases through structural destabilization.	[14,43]

Abbreviations: NET—Neutrophil extracellular trap (a chromatin-based structure released by activated neutrophils that promotes inflammation and coagulation), SPP1—Secreted phosphoprotein 1 (osteopontin) (a multifunctional protein expressed by macrophages and other cells, involved in inflammation, tissue remodeling, and angiogenesis).

**Table 7:** Platelet-derived extracellular vesicles as mediators of thromboinflammation in CAD and potential translational tools.

Biological Dimension	Description	Pathophysiological Consequence	Translational Relevance	References
Procoagulant surface properties of pEVs	Phosphatidylserine exposure on pEVs increases the efficiency of coagulation complex assembly and enhances thrombin generation.	Local thrombogenicity increases in areas of endothelial injury and plaque destabilization.	This mechanism supports evaluating pEVs as biomarkers of procoagulant activation.	[60,61,62]
Endothelial activation and increased leukocyte adhesion	pEVs can induce ICAM-1 and VCAM-1 expression and amplify pro-inflammatory programs in endothelial cells.	Barrier permeability increases and leukocyte influx into the lesion is enhanced.	This phenomenon supports using pEV profiles to monitor inflammatory activity.	[16,18,20,62]
Transfer of RNA regulators	pEVs carry microRNAs that modulate the expression of inflammation- and metabolism-related genes in vascular and myeloid cells.	Target-cell phenotypes are reprogrammed toward pro-inflammatory or pro-atherogenic states.	This mechanism provides a rationale for liquid biopsy concepts based on EVs and miRNAs.	[63,64,65]
Reinforcement of the platelet–neutrophil axis and NETosis	pEVs can stimulate NETosis, and NETs further enhance platelet activation and coagulation.	A feedback loop develops that increases both inflammation and thrombogenicity.	Disrupting this loop may represent a therapeutic target at the immunothrombotic interface.	[66,67,68]
Clinical relevance and metabolic modifiers	Levels of pEVs and other EVs correlate with microcirculatory parameters and event risk, and metabolic disorders influence their abundance and cargo.	EVs may reflect the intensity of atherosclerotic–thrombotic processes over time.	EVs may support risk stratification and monitoring of treatment response.	[62,69,70,71]

Abbreviations: EVs—Extracellular vesicles (membrane-bound particles released by cells that mediate intercellular communication), ICAM-1—Intercellular adhesion molecule 1 (an endothelial adhesion receptor that facilitates leukocyte binding and transmigration), miRNAs—Micro Ribonucleic acids (small non-coding RNA molecules that regulate gene expression post-transcriptionally), NETs—Neutrophil extracellular traps (web-like chromatin structures released by neutrophils during NETosis that promote inflammation and coagulation), pEVs—Platelet-derived extracellular vesicles (a subtype of EVs released from activated platelets with procoagulant and proinflammatory properties), RNA—Ribonucleic acid (a class of nucleic acids involved in gene expression and regulation), VCAM-1—Vascular cell adhesion molecule 1 (an endothelial adhesion molecule that promotes leukocyte recruitment to sites of inflammation).

**Table 8:** Clonal hematopoiesis as a modifier of the thromboinflammatory phenotype in CAD: mechanisms and implications.

CHIP-Associated Risk Element	Description	Biological Effect in the Vascular System	Potential Clinical Implication	References
Mutations in epigenetic regulator genes (DNMT3A, TET2)	Epigenetic CHIP mutations can enhance inflammasome activity and increase IL-1β production in myeloid cells.	Endothelial activation and leukocyte recruitment to the vessel wall increase.	Residual risk related to chronic inflammation persists.	[75,77]
JAK2 mutation and prothrombotic tendency	JAK2 mutations can promote heightened pro-inflammatory signaling and an increased propensity for thrombogenesis.	The potential for procoagulant reprogramming of myeloid responses increases.	Thrombotic event risk may increase in selected patient subgroups.	[76]
Clone size and risk heterogeneity	A larger clonal contribution and specific mutation types are associated with worse prognosis.	Pro-inflammatory myeloid programs may become more sustained and persistent.	This supports risk stratification and personalization of interventions.	[78,79]
Potential responsiveness to anti-inflammatory treatment	Because CHIP effects are largely mediated by cytokines, modulation of the IL-1β/IL-6 axis is being considered.	Endothelial activation and leukocyte recruitment may be reduced.	Anti-inflammatory therapies may provide greater benefit in high-risk populations.	[75,80]

Abbreviations: DNMT3A—DNA (deoxyribonucleic acid) methyltransferase 3A (an epigenetic regulator involved in DNA methylation), IL-1β—Interleukin-1 beta (a proinflammatory cytokine central to inflammasome signaling), IL-6—Interleukin-6 (a pleiotropic cytokine involved in inflammation and immune regulation), JAK2—Janus kinase 2 (a tyrosine kinase mediating cytokine receptor signaling and hematopoiesis), TET2—Ten-eleven translocation 2 (an epigenetic modifier involved in DNA demethylation).

**Table 9:** Therapeutic nodes in CAD immunothrombosis and the rationale for their targeting from a network perspective.

Therapeutic Node	Description	Mechanistic Rationale in CAD	Expected Biological Effect	References
IL-1β/IL-6 axis and downstream pathways	Inhibition of the IL-1β/IL-6 axis aims to reduce the amplitude of innate immunity–driven inflammation.	This axis integrates inflammatory signaling with endothelial activation and leukocyte recruitment.	Inflammatory activity within the plaque is expected to decrease and destabilization risk is expected to be reduced.	[81,83,84,85]
NLRP3 inflammasome	Inhibition of NLRP3 aims to limit the maturation of pro-inflammatory mediators and the persistence of innate immune activation.	The inflammasome represents a node that amplifies cytokine networks and links inflammation with atherosclerosis.	Feedback loops that sustain thromboinflammation are expected to be attenuated.	[81,83,85]
P-selectin/PSGL-1 interaction	Targeting the P-selectin/PSGL-1 pathway aims to reduce platelet–leukocyte aggregate formation.	These aggregates enhance leukocyte recruitment and activation and promote procoagulant activity.	Thromboinflammation is expected to decrease while preserving physiological hemostasis.	[83,84,85]
NET and PAD4-related mechanisms	Inhibiting NETosis or its downstream effectors aims to limit the formation of a procoagulant scaffold within the lesion.	NETs provide a matrix that amplifies thrombin generation and endothelial injury.	NET-dependent thrombogenicity is expected to be reduced.	[28,87]
EV and pEV axis	Interventions targeting extracellular vesicles aim to limit the transfer of pro-inflammatory and procoagulant cargo.	EVs transport lipid mediators and RNA that reprogram vascular and myeloid cells.	Intercompartmental signaling is expected to be weakened and amplification of thromboinflammation is expected to be reduced.	[88,89,90,91]
Spatially guided drug delivery	Spatial strategies aim to concentrate therapeutic agents within high-risk niches defined by microenvironmental signatures.	Plaque heterogeneity limits the efficacy of systemic therapies and facilitates the persistence of pathogenic niches.	Higher efficacy with a lower risk of adverse effects is expected.	[81,82,92,93]

Abbreviations: CAD—Coronary artery disease, EV—Extracellular vesicle (a membrane-bound particle released by cells that mediates intercellular communication and transfer of bioactive molecules), IL-1β—Interleukin-1 beta (a key proinflammatory cytokine involved in innate immune activation), IL-6—Interleukin-6 (a cytokine with central roles in inflammation and immune regulation), NET—Neutrophil extracellular trap (a DNA-based structure released by neutrophils that promotes inflammation and coagulation), NLRP3—NOD-like receptor family pyrin domain containing 3 (a key component of the inflammasome involved in activation of inflammatory responses), pEV—Platelet-derived extracellular vesicle (a subtype of EV released from activated platelets with procoagulant properties), PAD4—Peptidylarginine deiminase 4 (an enzyme that mediates histone citrullination and is essential for NET formation), PSGL-1—P-selectin glycoprotein ligand-1 (a leukocyte surface receptor that binds P-selectin and mediates platelet–leukocyte interactions).

## Data Availability

No new data were created or analyzed in this study. Data sharing is not applicable to this article.
